# Interaction between dietary fatty acids and genotype on immune response in Atlantic salmon (*Salmo salar*) after vaccination: A transcriptome study

**DOI:** 10.1371/journal.pone.0219625

**Published:** 2019-07-31

**Authors:** Adriana Magalhães Santos Andresen, Esmail Lutfi, Bente Ruyter, Gerd Berge, Tor Gjøen

**Affiliations:** 1 Department of Pharmacy, Section for Pharmacology and Pharmaceutical Biosciences, University of Oslo, Oslo, Norway; 2 Nofima (Norwegian Institute of Food, Fisheries and Aquaculture Research), Ås, Norway; National Cheng Kung University, TAIWAN

## Abstract

A pivotal matter to aquaculture is the sourcing of sustainable resources as ingredients to aquafeeds. Levels of plant delivered oils as source of fatty acids (FA) in aquafeeds have reached around 70% resulting in reduced levels of long-chain omega-3 polyunsaturated fatty acids (LC n-3 PUFA), such as eicosapentaenoic acid (EPA) and docosahexaenoic acid (DHA), in salmon fillet composition. EPA and DHA can modulate inflammation and immune response, so it is crucial to understand how fish immune response is affected by low LC n-3 PUFA diet and if this diet can have a detrimental effect on vaccine response. Atlantic salmon (*Salmo salar*) can produce EPA/DHA from α-linolenic acid (ALA) and this endogenous capacity can be explored to develop families with higher tolerance to low LC n-3 PUFA diets. Here we analyze innate and adaptive immune response in Atlantic salmon to a commercial vaccine after being fed low levels of EPA and DHA, and we also compare three strains of salmon selected by their endogenous capacity of synthesizing LC- n-3 PUFA. A total of 2,890 differentially expressed genes (DEGs) were identified (p-value adjusted < 0.1) when comparing vaccinated fish against control non-vaccinated. Gene ontology (GO) and KEGG analysis with 442 up/downregulated genes revealed that most DEGs were both related to immune response as well as part of important immune related pathways, as “Toll-like receptor” and “Cytokine-Cytokine interaction”. Adaptive response was also addressed by measuring antigen specific IgM, and titers were significantly higher than in the pre-immune fish at 62 days post-immunization. However, diet and strain had no/little effect on vaccine-specific IgM or innate immune responses. Atlantic salmon therefore display robustness in its response to vaccination even when feed low levels of LC n-3 PUFA.

## Introduction

Aquaculture is the fastest growing sector in food production worldwide and will soon provide more seafood than the global fish capture [1]. The concomitant need for aquafeeds, based on marine resources, will surpass the available supply in a few years and will therefore have to be replaced with sustainable plant or algal/microbial resources [2]. In 2016, total production of fish and other aquatic animals reached 170 million tonnes and aquaculture was responsible for 80 million tonnes [1]. This increase in aquaculture production, over the last 50 years, has made it possible for global fish consumption to reach 20 kg per capita in 2014 (compared to about 10 kg in the 1960s), and it is still increasing [1, 3]. To sustain aquaculture growth and ensure that it remains economically viable, without being detrimental to the environment, alternatives to fishmeal (FM) and fish oil (FO) based are constantly being studied and developed. A large fraction of fishery production, around 20 million tonnes in 2016, was not used for human consumption, but mainly for production of FM and FO [4]. The demand for aquafeed has grown more than expected and it is necessary to find other sources that can fulfill fish nutritional requirements to grow healthy and to provide high-quality fillet for human consumption [5, 6]. For the last decades much research has been done both aiming to develop new alternatives to fishmeal and fish oil [7, 8] as well as to understand the consequences of these feeds regarding fish health, optimum growth and flesh quality [9–11]. Fortunately, the transition from an aquafeed based on marine resources to a feed based on plant materials has been underway for many years already [12, 13]. Plant based ingredients are slowly taking over as the main raw materials in fish feed, and although these changes seems to be well tolerated by fish, an inevitable change in the lipid profile of the final product is also taking place [14]. Many studies evaluating the effects of plant based feeds on the growth and health of Atlantic salmon have been performed on relatively small fish in the freshwater phase [15, 16] and, in recent years, several studies have confirmed that a plant based diet can support growth and health in salmon throughout whole the production cycle [17]. The reported dietary requirement of n-3 FAs ALA, EPA and DHA of salmonids has been stated to range from 10 to 25 g/kg feed, depending on the species and experimental conditions [18]. Although these levels can sustain normal growth and health under laboratory conditions, there is a need for more information about the lowest levels during actual farming conditions where fish is subjected to environmental and farming stressors like changes in temperature, transport, infections and vaccination.

The effects of dietary lipids on immune functions are well documented in many vertebrate species [19–24]. Unsaturated fatty acids like EPA and DHA have, in many of these studies, been suggested to play an important anti-inflammatory role upon immune regulation during both infections and autoimmune diseases [25]. Several dietary studies, combined with analysis of immune function, have been performed in salmon but with no clear conclusions about dietary requirements [20, 26–31]. More studies investigating the effects of marginal levels of LC n-3 PUFA combined with different forms of stress, relevant to the farming conditions, are therefore needed. Since all farmed salmon today are vaccinated against multiple bacterial and viral diseases, more knowledge about the impact of feed on vaccination is required [32]. An additional strategy to enhance the sustainability of salmon farming is to selective breed animals with enhanced capacity for endogenous production of EPA and DHA from dietary alpha-linolenic acid. Such animals may have lower dietary requirements for the limiting raw marine ingredients in the fish feed industry. To test this hypothesis, we challenged two groups of salmon with such characteristics (low and high delta-6 desaturase) and a control group (standard non-selected strain) fed with different levels of n-3 PUFA, with a commercial vaccine. After vaccination, both innate and adaptive immune responses were measured by analysis of the head kidney transcriptome and quantification of specific antibody against vaccine antigen, respectively. Effects of diet and strain on these parameters were also compared.

## Materials and methods

### Animals, ethics and culture conditions

Experimental fish consisted of two Atlantic salmon genetic groups with different capacity to produce EPA and DHA in addition to control. The two experimental strains were selected for increased/decreased innate delta-6 desaturase expression (Hid6fad/Lowd6fad) [33], while control group were composed of standard production fish that was not selected for any specific phenotype (NS). Fish approximately 200 g in weight, were individually pit-tagged (PIT-tags, Passive Integrated Transponder, Biosonic) and maintained at the NOFIMA Sunndalsøra fish facilities at a 12L:12D photoperiod in 12 tanks (500 L, 36 fish/tank) in a temperature controlled recirculation system (10 ± 1 °C) and were fed four experimental diets in excess using automatic belt feeders until they reached average final weights of 400g (3 months). The experimental diets were designed to contain approximately same amounts of α-linolenic acid (ALA) and different ratios between EPA and DHA (Table 1). Diet 1 (ALA): only based on plant oil and no FO, with EPA and DHA constituting 0.1% of total FAs in this diet; diet 2 (EPA): supplemented with EPA rich FO, so that EPA was 3.4% and DHA 1% of total FAs in the diet; diet 3 (DHA): supplemented with a DHA rich FO so that DHA was 3.7% and EPA 0.6% of total FAs, and diet 4 (EPA/DHA): was supplemented with a mix of the two FOs in order to have equal amounts of EPA and DHA in the diet, these FAs were each 2.2% of total FAs. Diets 2, 3 and 4 were formulated to contain the same level of EPA + DHA, 4.4% of total FAs in the diets. All diets contained 47.3% protein, 24% lipid, 8.1% starch and were isoenergetic (21.4 MJ/kg).

**Table 1:** Total lipid content (% of total lipids) and fatty acid composition (% of total fatty acids) of experimental diets.

	ALA	EPA	DHA	EPA/DHA
**Saturated**	11.6	10.9	11.3	11.2
**Monounsaturated**	45.2	41.8	42.1	42.0
**Polyunsaturated**	42.0	45.2	45.3	44.9
**Total n-6**	23.0	22.5	22.5	22.5
**Total n-3**	19.1	22.8	22.8	22.4
**EPA**	0.0	3.4	0.6	2.2
**DHA**	0.1	1.0	3.7	2.3
**Total EPA/DHA**	0.1	4.3	4.4	4.5
**Lipid Content**	24.1	24.6	24.6	24.7

The vaccination experiment with Atlantic salmon was conducted in compliance with the national regulation for use of experimental animals (FOR-2015-06-18-761, §2-f, corresponding to Directive 2010/63/EU Article 1, section 5f) and Norwegian Food Safety Authority FOTS approval ID 8378 of the experiment.

### Vaccination

Prior to vaccination, 400g fish were starved for 24 hours. Plasma samples from three individual fish in each tank were obtained as pre-immune controls (36 samples) for ELISA analysis. The experimental fish were anesthetized with 60 mg/L tricaine metanesulfate (MS-222, Finquel, Argent Chemical Laboratories, Redmond, WA, USA) and 15 fish from each dietary group, 60 fish in total, were vaccinated with 50 μl of a commercial hexavalent vaccine (ALPHA JECT micro 6, PHARMAQ, NORWAY) according to the manufacturer’s procedure. Six fish were injected with 50 μl PBS and used as controls for the overall effect of vaccination. After injection, the fish were kept in separate tanks (per feed group) until the first sampling, 24 hours after vaccination. Head kidney from twelve fish, from two groups (Hid6fad and NS), were sampled in RNAlater (Thermo Fisher Scientific Inc., Waltham, MA, USA) for tissue preservation (Lowd6fad were not sampled for RNA isolation). Each strain group had three fish from each experimental diet (a total of 24 fish). Head kidney from control fish, mocked vaccinated (injected with PBS), was also sampled (six fish—three NS and three Hid6fad). Fish that were not sacrificed for RNA isolation at 24 hours after vaccination were transferred to a common tank and fed diet 4, which is close to a commercial diet in composition, for the next two months. During this period fish were kept under comparable environmental conditions as before vaccination (salinity and water temperature). A flowchart of the study design is provided in supplementary material (Fig A in S1 Text).

After 62 days post-immunization, fish were anesthetized with MS 222. Twelve fish from each strain (Hid6fad/Low6fad and NS) were sampled. Each strain group had three fish from each experimental diet (a total of 36 fish). A 2–3 ml blood sample from each fish was obtained in EDTA vacutainers (Becton & Dickinson, USA) before sacrifice and tissue sample collection. Blood were centrifuged at 2000 x g for 15 minutes and plasma was carefully removed. Tissue samples from head kidney, heart, spleen and liver were placed in cryotubes containing RNAlater. All the samples, plasma and tissue, were stored at—80 °C before analysis.

### Total RNA isolation and sequencing

Total RNA was extracted using Rneasy Mini Kit (QIAGEN, Hilden, Germany) according to the manufacturer’s tissue protocol. A step for removal of genomic DNA was included: 15 min incubation, at room temperature, with Dnase I (Rnase-Free Dnase Set, QIAGEN, Hilden, Germany). Total RNA was eluted in 50 μl Rnase-free distilled water and concentration was measured using PicoDrop Pico100 (PicoDrop Technologies, Cambridge, UK). The Norwegian Sequencing Centre (NSC) verified the RNA quality with Agilent 2100 Bioanalyser (Agilent, USA), and performed library preparation, using TruSeqTM Stranded mRNA Library Prep Kit (Illumina Inc., San Diego, USA). Libraries were then sequenced on Illumina HiSeq 4000 sequencer, where 150-bp paired-end reads were obtained.

### Validation of RNA sequencing (RNA-seq) by quantitative PCR (qPCR)

RNA was reverse transcribed to cDNA using high-capacity RNA-to-cDNA kit (Applied Biosystems Inc., United States), following manufacturers protocol. qPCR was performed in 96-well plates on LightCycler 480 using SYBR Green Master Mix (both from Roche Diagnostics, Basel, Switzerland). Cycling conditions were: 95 °C for 5 minutes, 40 cycles of 10 s at 95 °C, 10 s at 60 °C and 10 s at 72 °C. Melting curve was measured at 95 °C for 5 s followed by 1 min at 65 °C. All qPCR experiments were performed using three biological and two technical replicates. Cycle threshold (Ct) values were obtained and used to calculate correlation. For calculation of relative expression levels, delta-delta Ct method was used [34]. 18s and ef1a were used as reference genes [35]. Primers used are listed in Table 2.

**Table 2:** qPCR primers.

Genes	Direction	Sequence 5′→3′	Accession Number	Amplicon	Reference
*ef1a*	F	CACCACCGGCCATCTGATCTACAA	AF321836	77	[36]
	R	TCAGCAGCCTCCTTCTCGAACTTC			
*18S*	F	TGTGCCGCTAGAGGTGAAATT	AJ427629.1	61	[36]
	R	GCAAATGCTTTCGCTTTCG			
*actb*	F	GCTGACAGGATGCAGAAGGAAA	AF012125.1	214	[35]
	R	CGGCGGTGCCCATCT			
*ccl4*	F	TGCACAAAGGTCTCCAAGCA	XM_014191808.1	84	*
	R	AGCATTGACACAGGGAAGGG			
*grp78*	F	ACGGCATCTTGCGCGTCACA	NM_001141642.1	242	[26]
	R	CAGCTTGCCGCCCAGCTTCT			
*il1β*	F	GGAGAGGTTAAAGGGTGGCG	AY617117	51	[37]
	R	TCCTTGAACTCGGTTCCCAT			
*socs3*	F	GGGAAGGCAGCAACATGAGT	XM_014202622.1	84	*
	R	TTTTGGTTGGCAGCCTGTTG			
*fadsd6_a*	F	TCCCCAGACGTTTGTGTCAGATGC	AY458652	171	[38]
	R	GCTTTGGATCCCCCATTAGTTCCTG			
*fadsd6_b*	F	TGACCATGTGGAGAGTGAGGG	GU207400	249	[38]
	R	AACTTTTGTAGTACGTGATTCCAGCT			
*fadsd5*	F	AGAGGCACTCCCACAGAAGC	AF478472	51	[39]
	R	AGACCTTCCTGTCGATGACCA			
*hsp90ab*	F	ACACGGTGTTGGGTTGGTTT	AF135117.1	51	[37]
	R	CCATGCAGCGTGCATGTTAT			
*atg3*	F	CCTTCTCCTCTTCCCCAGAC	BT057906.1	173	*
	R	TAATTCCGTAAAAGGCACGG			

* Primers designed for this study. *ef1a—elongation factor 1 alpha*, *18s—18S ribosomal RNA*, *actb—actin beta*, *ccl4—c-c motif chemokine ligand 4*, *grp78—glucose regulated protein 78*, *il1β—interleukin 1 beta*, *socs3—suppressor of cytokine signaling 3*, *fadsd6* (_a/_b)—*delta-6 fatty acyl desaturase* (_a/_b), *fadsd5*—*delta-5 fatty acyl desaturase*, *hsp90ab—heat shock protein 90 alpha-b* and *atg3—autophagy related 3*.

### Enzyme Linked Immunosorbent Assay (ELISA)

ELISA measuring salmon IgM antibodies specific for one of the vaccine antigens, *Aeromonas salmonicida* bacterin, was performed using 96 well Maxisorp microtiter plates (Sigma-Aldrich, St. Louis, USA). Plates were first coated with 100 μl of PLL (5 μg/ml poly-L-lysine in PBS) for 1 hour at room temperature. After three washes with washing buffer PBST (phosphate buffered saline, pH 7.4 with 0.01% Tween 20), plates were coated overnight with bacterin solution (inactivated bacteria diluted to OD_600 nm_ = 0.5 in 2% NaCl) at 4 °C. Plates were washed three times and blocked with PBST containing 5% dry milk for 2 hours. After another three washes, plates were ready for IgM ELISA. The assay was first validated with plasma from control (mix of 10 samples from non-vaccinated fish) and test (mix of 10 samples from vaccinated fish). Bacterin coated plates were incubated overnight with 100 μl 2-fold dilution (in PBST with 1% dry milk—PBSTM) of test and control plasma. After washing thrice, all wells were incubated with 100 μl mouse monoclonal anti-trout/salmon IgM (4C10) [40] diluted 1:3,500 in PBSTM, for 2 h at room temperature, which was followed by three washes and incubation with anti-mouse IgG-HRP (dilution 1:500 in PBSTM; Sigma-Aldrich, St. Louis, USA) before development with 1-step slow TMB substrate (Thermo Fisher Scientific Inc., Waltham, MA, USA) according to the manufacturer’s instructions. The absorbance signal was measured with a Clariostar plate reader (BMG Labtech, Offenburg, Germany). After validation of the assay (Fig B in S1 Text) all samples were analyzed using the same protocol. Data were expressed as optical density (OD), adjusted for background.

### Bioinformatics and statistics

Fastq files containing reads from the RNA-seq were mapped to Atlantic salmon genome (GCF_000233375.1_ICSASG_v2_genomic.fna), using the HISAT-Stringtie pipeline [41], and transcripts were assembled using the existing Atlantic salmon annotation file (GCF_000233375.1_ICSASG_v2_genomic.gff) as input. Both files were downloaded from NCBI (Annotation release 100). After mapping and assembly of full and partial transcripts, R package Ballgown (version 2.12.0) was used to quantify differential expression between samples. After creating tables with transcript FPKM (Fragments Per Kilobase Million) mapped to genes (using the *gexpr* function), two rounds of analyses were performed. First the effect of vaccination on salmon head kidney transcriptome was evaluated by dividing the samples into only two groups: control and vaccinated (ignoring strain and diet). Output from this analysis was only used to confirm the effect of vaccine (differential expression of immune related transcripts, enrichment of gene ontology terms and KEGG pathways related to innate immunity). This analysis calculates fold change in expression between vaccinated and control. In the second analysis, only vaccinated fish were included, since we wanted to evaluate the effects of strain and diet on the outcome of vaccination with respect to these variables and their interaction. Genes with adjusted p-value (q-value—qval) below 0.1 were regarded as differentially expressed genes (DEG).

Pre- and post-vaccination antibody titers were compared using T-test whereas differences between strains and dietary groups were calculated by one or two-way ANOVA using R statistical software. For multiple comparison tests, with the qPCR results from liver samples, TukeyHSD (Tukey Honest Significant Differences) was applied and qval < 0.05 was considered significant. All scripts for exploratory plots and expression analysis are available in S1 Text (Table A and Fig C-H), S1 and S2 Scripts.

## Results

### Head kidney transcriptome

RNA-sequencing was used to analyze head kidney transcriptome at 24 hours after vaccination. At this time point, innate immune responses to the vaccine have been initiated both locally, at the injection site (abdominal), and at distant sites rich in immune cells (spleen and head kidney) [42]. The number of sequenced reads from each sample varied from 15 to 35 million achieving an average alignment rate of 83% (range 63–87%) of the reads mapped to Atlantic salmon genome (Table B in S1 Text). To estimate the expression levels of the genes we used the R package Ballgown [43]. Using the *gexpr* function, a gene table was generated containing 23,943 expressed genes, after cleaning for low counts genes (row mean > 1 FPKM).

### Effect of vaccination

**Fig 1:**
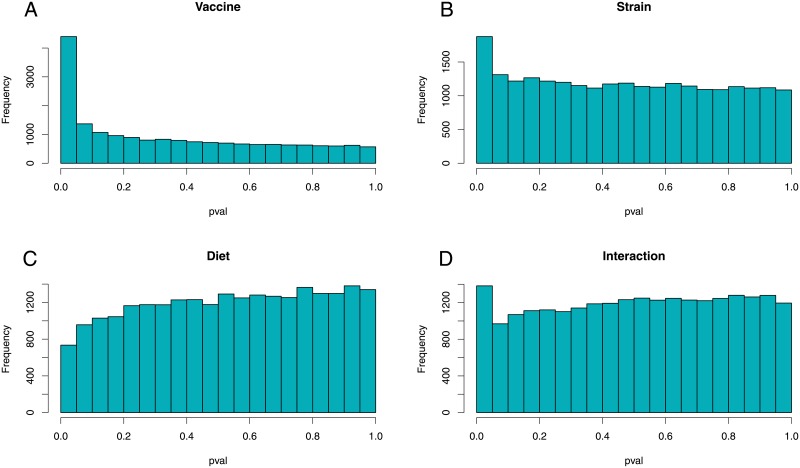
P-value distribution histogram. Ballgown *stattest* function used to specify effect of treatment and covariates. (A) Control fish (n = 6) versus vaccinated fish (n = 24). (B) Vaccinated fish only and strain as covariate (n = 24). (C) Vaccinated fish only and diet as covariate (n = 24). (D) Interaction analysis of diet and strain in vaccinated fish (n = 24).

**Fig 2:**
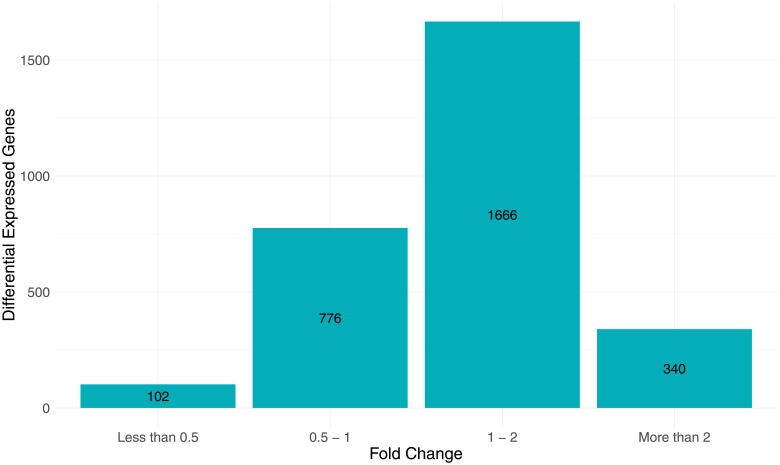
Number of genes differentially expressed in head kidney induced by vaccination, one day after immunization. Control fish (n = 6) versus vaccinated fish (n = 24).

First we analyzed the effect of the vaccine on head kidney transcriptome. We divided 30 fish into two groups: control non-vaccinated fish (n = 6) and vaccinated fish (n = 24) without taking into consideration different strains and diets. Running *stattest* function of Ballgown resulted in a table containing fold change and qval between vaccinated and control fish. After merging the gene expression list (containing 23,943) with a salmon annotation list, 5,227 genes were excluded due to missing gene ID (gene model do not match gtf file). A unique gene ID is necessary to run both gene ontology analysis as well as KEGG pathway analysis. The result was a table containing 18,716 genes expressed in head kidney (S1 Table). Fig 1 shows p-value distribution of multiple analysis using different covariates. When a p-value histogram, like on Fig 1A, 1B and 1D, displays a cluster of p-values near zero it indicates that some genes expression levels changed significantly after vaccination, showing a clear effect. On Fig 1C, where we test diet as a covariate, we observe an approximately uniform distribution of p-values, indicating that the effect of vaccination on gene expression is not affected by diet alone. A total of 2890 DEGs (S2 Table) were considered significant (qval < 0.1) and, among those genes, 340 were upregulated by 2-fold or higher and 102 genes were downregulated by fold change < 0.5 (Fig 2).

**Fig 3:**
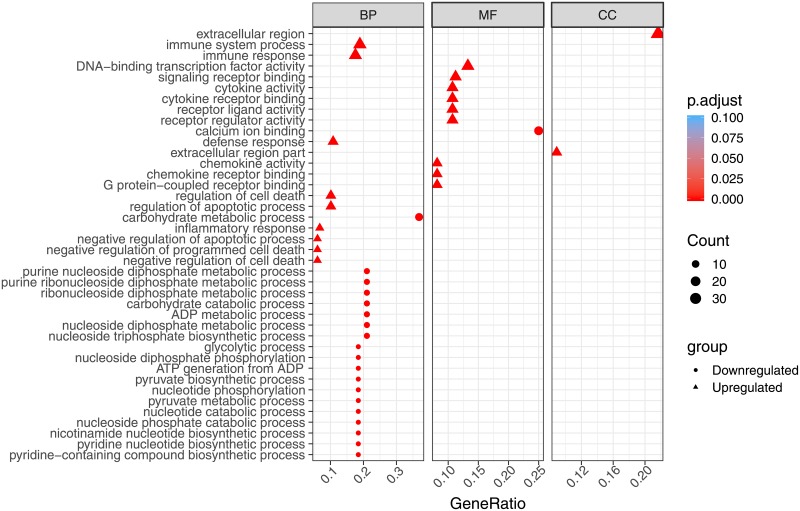
Gene ontology enrichment analysis of the differentially expressed genes from vaccinated fish comparing to control fish. GO annotation based on *Salmo salar* OrgDb object. BP—Biological Processes, MF—Molecular Function and CC—Cellular Components. Color gradient from red to blue, where red indicates high enrichment (low p.adjust) and blue indicates low enrichment (high p.adjust). Dot size corresponds to count (number of DEGs in each term).

**Fig 4:**
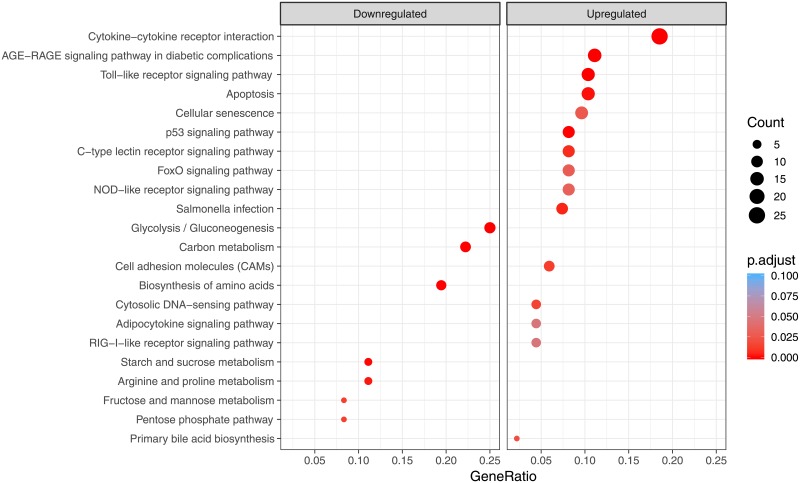
KEGG analysis of the differentially expressed genes from vaccinated fish comparing to control fish. Color gradient from red to blue, where red indicates high enrichment (low p.adjust) and blue indicates low enrichment (high p.adjust). Dot size corresponds to count (number of DEGs in each pathway).

**Fig 5:**
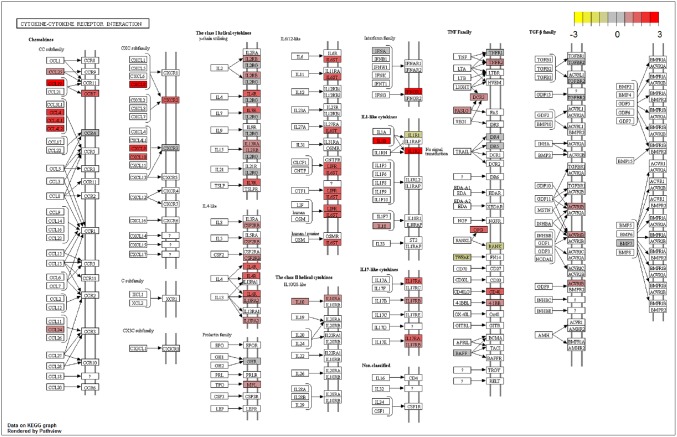
KEGG pathway map. Cytokine-cytokine receptor interaction network map for DEGs of vaccinated fish. Red squares represent upregulated genes, while yellow shows downregulated genes. Grey are genes with lower expression levels. Fold change values are represented in log2 scale.

From 442 up/downregulated DEGs, we identified enriched terms from the different GO categories (S3 Table): 145 biological processes (BP), 25 molecular function (MF) and 4 cellular components (CC), using R package clusterProfiler [44]. GO terms with a q-value lower than 0.05 were considered significant. Over-representation analysis of gene ontology biological process category showed that upregulated GO terms were related to immune response (as “Immune system process” and “Inflammatory response”) and apoptotic processes. Among downregulated genes we identified functional groups connected with metabolic processes, like carbohydrate and pyruvate metabolic processes (Fig 3), showing that vaccination not only can activate immune related pathways, but it may also have an impact on more general metabolic processes. When MF GO analysis was performed, mostly upregulated enriched terms (22 out of 24) were identified. “Cytokine activity”, “Chemokine receptor binding”, “Signaling receptor binding” were between those over-represented categories. Only two categories were identified in the downregulated genes: “calcium ion binding” and “MAP kinase activity”. CC analysis did not result in many significant GO terms, and we were only able to identify four categories, all four upregulated and related to extracellular region (reflecting cytokine secretion). KEGG pathway enrichment analysis was performed to identify to which pathways the significant DEGs belonged (S4 Table). When KEGG data from salmon was merged with our differentially expressed genes list, 1,957 genes (out of 2,890) were assigned to a KO (KEGG Orthology) identifier. After filtering (qval < 0.05), 22 significantly enriched pathways containing 95 unique DEGs were identified (Fig 4). Important pathways related to innate immune response, as Toll-like, NOD-like and RIG-I-like receptor signaling pathways, were enriched. As *enrichGO* function do not take in consideration level of expression, we used pathview R package to draw some of the KEGG maps showing both genes that are present as well as their expression levels (Fig 5).

### Effect of strain and dietary PUFA after vaccination

**Fig 6:**
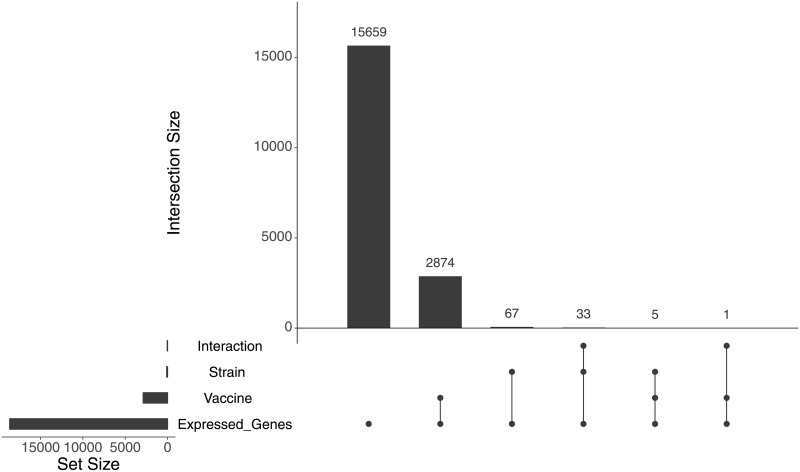
UpSet plot showing overlapping of genes identified by each of the different analyses. The bars show the overlap between the indicated motifs below: Expressed_Genes (all genes expressed in head kidney), Vaccine (DEGs in vaccinated fish), Strain (only vaccinated fish with strain as covariate) and Interaction (interaction of strain and diet in vaccinated fish only).

As the main purpose of this study was to test if diet and/or strain could influence the immune response induced by vaccination, we also analyzed DEGs excluding the control group (non-vaccinated fish), comparing only vaccinated fish. We identified 105 genes that were significantly differentially expressed and affected by strain (S5 Table). Among those genes, 27 and 11 were upregulated or downregulated more than twofold, respectively. Genes like galectin-9, *cmf35-like molecule 8* and *Ig Kappa chain V* were upregulated, while glutaredoxin-1, phosphatase and actin regulator-3 and fructose-1,6-bisphosphatase 1-like were downregulated. Not a single significant gene was identified when the effect of diet alone was tested in vaccinated fish. However, although no DEGs were found when testing for diet effect alone, an interaction model, including strain and diet in the vaccinated group, was used to determine if an interaction between the variables was present. As a result, 34 DEGs were identified (Table 3). When comparing these genes sets with the set of genes affected by vaccination, 100 DEGs were identified that were not affected by vaccination. Among these 100 genes, 67 were only influenced by strain and 33 appear on both strain and interaction lists (Fig 6).

**Table 3:** List of DEGs affected by the interaction of strain and diet in vaccinated fish (n = 24).

GeneID	Description	pval	qval	mean
106587643	tripartite motif-containing protein 29-like	2.60E-04	9.43E-02	63.61
100196218	Glutaredoxin-1	1.22E-05	1.33E-02	31.73
106580299	polyubiquitin-like	2.79E-04	9.68E-02	23.01
106597012	Ig kappa chain V-IV region JI-like	1.79E-04	8.55E-02	19.61
106578920	uncharacterized LOC106578920	2.09E-04	8.73E-02	12.86
100194720	uncharacterized LOC100194720	4.83E-05	3.40E-02	12.02
106576033	leukotriene A-4 hydrolase-like	3.05E-04	9.81E-02	10.89
106575428	Ig kappa chain V region K16-167-like	2.89E-04	9.79E-02	10.36
106580350	selenoprotein M-like	2.17E-04	8.79E-02	9.17
100194722	uncharacterized LOC100194722	2.70E-04	9.64E-02	7.17
106561467	uncharacterized LOC106561467	2.54E-06	5.07E-03	5.87
106590951	natterin-like protein	1.94E-04	8.73E-02	5.27
106602317	probable E3 ubiquitin-protein ligase HERC6	5.49E-06	8.07E-03	5.12
106600446	C-C motif chemokine 4-like	5.46E-05	3.63E-02	4.38
106591921	probable E3 ubiquitin-protein ligase HERC6	2.48E-04	9.43E-02	4.08
106575836	protein N-lysine methyltransferase METTL21A-like	2.26E-05	2.07E-02	3.91
106599799	uncharacterized LOC106599799	1.49E-05	1.55E-02	3.64
100194614	c20orf149 protein	1.11E-05	1.31E-02	3.53
106593599	uncharacterized LOC106593599	5.14E-05	3.52E-02	3.53
106596688	uncharacterized LOC106596688	3.05E-04	9.81E-02	3.32
106583825	phosphatase and actin regulator 3-like	1.60E-07	1.27E-03	3.29
106568356	fructose-1,6-bisphosphatase 1-like	1.07E-04	5.97E-02	3.05
106576451	uncharacterized LOC106576451	2.54E-06	5.07E-03	2.79
106584776	guanine nucleotide exchange factor for Rab-3A-like	8.37E-06	1.05E-02	2.21
106580462	tyrosyl-DNA phosphodiesterase 2-like	2.17E-05	2.07E-02	2.1
106596610	phosphatase and actin regulator 3-like	2.48E-07	1.49E-03	2.03
106591664	carcinoembryonic antigen-related cell adhesion molecule 5-like	3.73E-05	2.88E-02	2.03
106566659	monoacylglycerol lipase ABHD6-like	2.54E-04	9.43E-02	1.7
106588726	tyrosine-protein phosphatase non-receptor type 12-like	3.48E-05	2.78E-02	1.54
106589432	E3 ubiquitin-protein ligase TRIM39-like	2.01E-04	8.73E-02	1.52
106606801	CMRF35-like molecule 8	2.95E-04	9.79E-02	1.45
106577870	basement membrane-specific heparan sulfate proteoglycan core protein-like	9.23E-05	5.39E-02	1.32
106595461	putative RNA exonuclease NEF-sp	2.22E-06	5.07E-03	1.26
106591787	uncharacterized LOC106591787	2.05E-04	8.73E-02	1.23

### Effect of diet and strain on vaccine-specific antibody response

**Fig 7:**
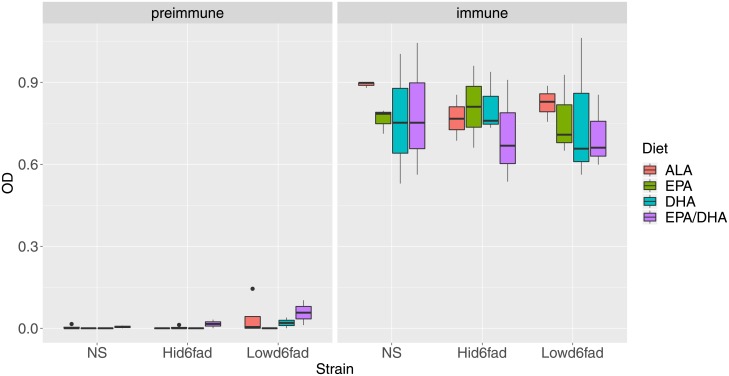
ELISA plot. Comparison of IgM titers in fish 62 days after immunization. Plasma from preimmune (n = 36) and vaccinated fish (immune, n = 36) were used to perform ELISA assay. Data expressed as OD adjusted for background. T-test used to calculate difference between immune and preimmune and ANOVA for multiple comparison between each variable (strain and diet).

Although the 24 h post-immunization (pi) head kidney transcriptome data may reveal alterations on innate immune responses, a better surrogate variable for immune function perturbations is the generation of vaccine-specific antibodies at 62 days pi. To test if dietary PUFA and/or strain affected vaccine-specific antibody responses, ELISA was performed on salmon plasma from fish in all groups. As can be seen from Fig 7 we observed a robust overall increase (from 0.01 ± 0.02 to 0.79 ± 0.14 OD_450nm_) in ELISA signal in the vaccinated fish (t-test, p < 2.2 x 10^−16^). However, when the effects of strain and dietary PUFA were tested, no significant differences were found between the groups (ANOVA, p = 0.78 and 0.80 for diet and strain, respectively).

### qPCR

**Fig 8:**
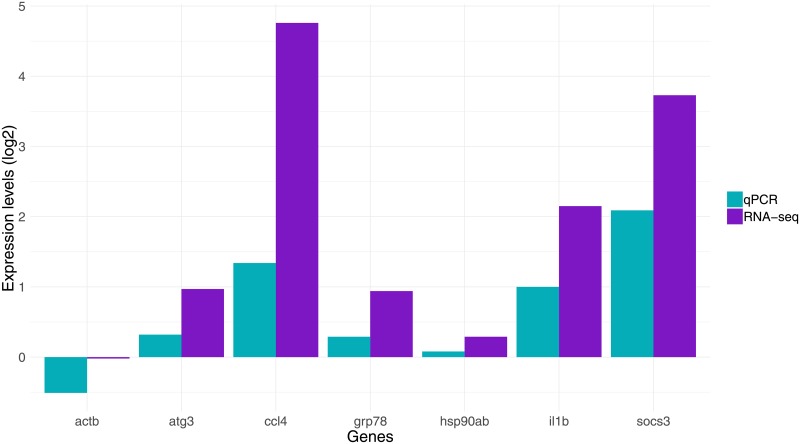
Comparison of qPCR and RNA-seq. Bar plot showing similar pattern of down/upregulation for tested genes on both analyses. Fold change values are represented in log2 scale from both RNA-seq and qPCR. 18s and ef1a were used as reference genes to calculate gene expression levels for qPCR data from three biological and two technical replicates.

qPCR was performed to validate RNA sequencing analysis and to assay gene expression of delta desaturase genes. For the validation, selected genes were chosen and qPCR was performed. Supplementary Fig A7 in S1 Text shows a strong correlation between RNA-seq and qPCR results. Although the levels of expression (Fig 8) are not identical, they do show a clear trend where genes display same pattern of downregulation/upregulation in both types of analyses.

**Fig 9:**
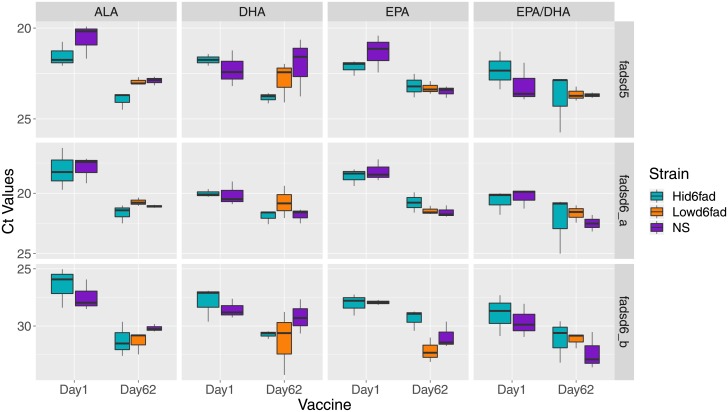
qPCR of liver samples from day 1 and day 62 after immunization. Box plot showing expression levels of three desaturase genes, fadsd5, fadsd6_a and fadsd6_b. This assay was performed with three biological and two technical replicates. TukeyHDS test was used to compare all the variables (strain/diet/time) against each other, and qval < 0.05 was considered significant (significant comparison shown in Table 4).

To assay the expression levels of delta desaturase genes in the different salmon strains, we performed qPCR analysis of liver samples from both day 1 and 62 after vaccination (when plasma was sampled for ELISA assay). Primer pairs for *delta-6 fatty acyl desaturase A* (fadsd6_a), *delta-6 fatty acyl desaturase B* (fadsd6_b) and *delta-5 fatty acyl desaturase* (fadsd5) were used to quantify the expression of these enzymes. Fig 9 shows the expression levels of the different desaturase genes at the end of the experimental feeding period (day 1) and after two months on the same diet (day 62). After vaccination all groups received diet 4 (EPA/DHA) which was closer to commercial feed, containing higher amounts of EPA/DHA compared to the other three diets (ALA, EPA and DHA). The general trend was a reduction in expression of desaturase transcripts when fish received higher levels of LC n-3 PUFA in the diet. In Table 4, we show the comparison between the three variables (strain/diet/time) that are statistic significant (qval < 0.05) and only in the same dietary group. Comparisons that are not shown in the table were not significant. Hid6fad fish fed ALA diet showed significant difference in expression of fadsd6_a and fadsd6_b, where the expression of these two genes was higher on day 1. Fish from all dietary groups showed a general trend (but q > 0.05) of desaturase downregulation upon transfer to high level PUFA diet.

**Table 4:** Comparison between strain, diet and day after vaccination.

Genes	Comparison	Difference	Lower	Upper	qval
fadsd6_a	Hid6fad:ALA:Day62 x Hid6fad:ALA:Day1	3.57	0.44	6.70	1.18E-02
fadsd6_a	Hid6fad:ALA:Day62 x NS:ALA:Day1	3.73	0.60	6.86	6.69E-03
fadsd6_a	NS:ALA:Day62 x NS:ALA:Day1	3.17	0.04	6.31	4.42E-02
fadsd6_a	Lowd6fad:EPA:Day62 x NS:EPA:Day1	3.26	0.12	6.39	3.39E-02
fadsd6_a	NS:EPA:Day62 x NS:EPA:Day1	3.37	0.24	6.51	2.31E-02
fadsd6_b	Hid6fad:ALA:Day62 x Hid6fad:ALA:Day1	4.81	0.44	9.17	1.76E-02
fadsd6_b	Lowd6fad:ALA:Day62 x Hid6fad:ALA:Day1	4.91	0.54	9.27	1.38E-02
fadsd5	Hid6fad:ALA:Day62 x NS:ALA:Day1	3.35	0.71	5.98	2.72E-03

Significant differences in desaturase expression comparing all strain and dietary groups (qval < 0.05) after performing TukeyHSD test on the Ct values from qPCR (Fig 9). Liver samples from day 1 and day 62 post-immunization fish were used (three biological and two technical replicates).

## Discussion

With the purpose of testing if different amounts of EPA and DHA in the feed could affect the immune response after vaccination, we tested four different diets containing different levels of EPA and DHA alone or in combination. Vegetable oils lack long-chain n-3 PUFA, as EPA and DHA, however, they are rich in linoleic acid (LA, 18:2 n-6) and alpha linolenic acid (ALA, 18:3 n-3) that can be converted to arachidonic acid (ARA, 20:4 n:6) and EPA/DHA, respectively [45]. As the ratio of omega 6 and omega 3 oils is an important health factor [46] an alternative strategy would be to choose oils that have a higher percent of ALA, as linseed oil, to take advantage of salmons endogenous capacity to produce EPA/DHA when fed diet where fish oil is replaced by vegetable oil [8, 47].

Our analysis, comparing head kidney transcriptome in vaccinated fish, using diet as a covariate, did not reveal any diet-related effects on gene expression. Divergence is found in literature about the effect of diet, levels of n-3 fatty acids, and resistance against infections and immune response. While Thompson et al. (1996) [48] reported that Atlantic salmon fed low ratios of n-3/n-6 PUFA were less resistant to infection, Gjøen et al. (2004) [49] observed no detrimental effects of low levels of EPA and/or DHA on salmon immune response and on susceptibility to *Aeromonas salmonicida* infection. In 2012, Zuo and collaborators [50] demonstrated that large yellow croaker (*Larmichthys crocea*) fed higher ratio of DHA/EPA had improvement in growth and enhanced protection against parasite infection. Diets with different amounts of camelina oil replacing fish oil did not result in strong changes in spleen gene expression profile or anti-viral immune responses in Atlantic cod (*Gadus morhua*) [51]. Caballero-Soares et al. (2017) [30] found no significant difference in response against poly I:C among fish receiving different diets containing fish oil or vegetable oil. However, higher transcript levels of some genes involved in antiviral immune response, such as *tlr3*, *isg15b* and *irf1b*, in Atlantic salmon fed plant-based feed were observed, suggesting that a plant-based diet may even enhance immune response. Comparison with those studies is difficult and it must be done with caution. Different levels of essential fatty acids, sources of protein, amounts of micronutrients as well as experimental conditions, can all have a profound impact on the results.

Another approach to reach desirable muscle deposition of LC n-3 PUFA and better conversion efficiency of ALA into EPA and DHA would be to exploit phenotypic and genotypic traits within salmon families. As stated before, Atlantic salmon is a net producer of EPA/DHA and can convert ALA into long-chain fatty acids. Variation between individuals in the ability to maintain higher levels of n-3 long-chain PUFA in the muscle have been reported and shown to be highly heritable [52–54]. In this study we compared vaccine response in three different strains of salmon selected for their capacity to produce long-chain PUFAs: a non-selected strain and two strain denoted Hi/Low-d6fad with higher/lower relative capacity for endogenous PUFA synthesis. The genetic explanation for these phenotypic differences are currently under investigation, but genetic analyses of these families revealed that when fish were fed moderate levels of plant oil during early life stages, there was an increased capacity of EPA and DHA synthesis in the high-desaturase group when comparing to the low-desaturase group [33]. When testing expression of different delta desaturase genes we observed that when fed diet with low levels of EPA and DHA (ALA diet) both Hid6fad and NS strains showed a higher expression of the tested genes (fadsd6_a, fadsd6_b and fadsd5). That has also been shown by other groups where fish fed vegetal oil based feed expressed higher levels of desaturase genes than fish receiving feed containing FO [55–57]. Higher expression do not mean higher levels of FA in the muscles, and the regulation of n-3 FA bioconversion pathway is very complex and can be affected by several factors including different regulation of the various gene copies and even temperature [38, 58].

For the RNA-seq analysis, we omitted the Lowd6fad salmon strain, as this was regarded as a less likely candidate for a future production strain. When analyzing only vaccinated fish, strain did have an impact in the expression of some immune relevant genes. Genes involved in immune response, like *lgp2* [59], Ig-kappa chain V-III region (igkv3-20) [60], galectin-9 [61] and *cmrf35* [62], were upregulated (fc > 2). Some genes from the TRIM family, *trim39*, *trim25* and *trim16* were also upregulated, but with a lower level of expression (fold change between 1 and 2). Genes belonging to TRIM family are involved in many processes like cellular proliferation, apoptosis, intracellular signaling and innate immunity. Up to now, more than 80 TRIM proteins have been identified in humans and some of these proteins are also involved in antiviral response [63, 64]. Gack et al. (2007) [65] reported *trim25* as being essential for RIG-I signaling. Jørgensen et al. (2008) [66] analyzed difference in gene expression between early mortality (EM) and late mortality (LM) in Atlantic salmon challenged *Salmon isavirus*. They found higher levels of Ig-kappa genes in EM fish, which are involved in B-lymphocyte maturation and humoral immunity, suggesting that expression of these Ig-kappa genes can lead to protection against pathogens. Although our results show a weak effect of strain on immune gene expression, some of those upregulated genes are involved in immune response and it may imply that fish with this specific genotype could mount a stronger response after vaccination.

Despite of the fact that diet alone had no significant effect on the head kidney transcriptome of vaccinated salmon, some genes were altered by the interaction of strain/diet. Leukotriene A-4 hydrolase-like (*lta4h*), *trim39*, *cmfr35* and *c-c motif chemokine 4-like* (*ccl4*) were all significant when tested for interaction. Lta-4 hydrolase is an enzyme that catalyzes the final step in the biosynthesis of Ltb4, and it is derived from the metabolism of polyunsaturated fatty acids like ARA [67]. Ltb4 is a proinflammatory mediator capable of recruiting and activating different immune cells, including neutrophils, and because of its strong chemoattractant activity, it is also involved in inflammatory and allergic disorders [68, 69]. Our results show that Hid6fad salmon kidney cells expressed lower levels of *lta4h* compared to the standard non-selected group. Significant lower levels were found in Hid6fad groups fed EPA and EPA/DHA combined. EPA inhibit ARA metabolism by substrate competition in this pathway, suppressing Ltb4 formation and, consequently, suppressing inflammation [70]. Another gene affected by strain/diet interaction was *ccl4*, where Hid6fad fish from dietary groups DHA, EPA and EPA/DHA showed higher expression levels of this chemokine. Hsu et al. (2013) [71] showed increased levels of *ccl4* expression in orange-spotted grouper (*Epinephelus coioides*) when stimulated with LPS or poly I:C and suggested that Ccl4 may enhance inflammatory reactions and trigger Th1 response leading to increased resistance against pathogens. Zang et al. (2017) [72] also showed upregulation of *ccl4* expression in large yellow croaker (*Larimichthys crocea*) after a trivalent bacterial vaccine indicating activation of TLR5M pathway. In mammals, studies showed that blocking CCl4, together with CCl3, eliminated most of the contribution of Cd4+ T-cell help to long-term Cd8+ T-cell memory [73, 74]. Our results show that interaction of diet and strain affected some immune related genes which may have an effect on the immune response after vaccination.

The main hypothesis to be tested in this study was that low levels of dietary or endogenous production of long-chain n-3 omega fatty acids, like EPA and DHA, change the specific antibody response to vaccination in Atlantic salmon. First, we analyzed the effect of vaccination on head kidney transcriptome without taking strain or diet into consideration, only to confirm the vaccine effect on head kidney transcriptome. Innate immune system is the first line of defense and considered the dominant system in combating pathogens in fish. It is highly conserved and constituted by lysozymes, complement, lectins, interferon, and pattern recognition receptors (PRR) among others [75, 76]. The list of upregulated genes by vaccination included different chemokines (*ccl4*, *cxc11* and *cxc19*), *socs3*, *hs90ab* and interleukins (*il1β*, *il8*, *il17ra*). All these genes have been shown by others to be affected by either vaccination or infection in fish [72, 77–81]. Gene ontology analysis revealed upregulation of functional terms involved in immune response. “Immune system process”, “immune response”, “response to stress” and “inflammatory response” were some of the overexpressed functional terms. KEGG analysis also showed significant enrichment of important pathways like “RIG-I receptor signaling pathway”, “Toll-like receptor signaling pathway” and “C-type lectin receptor signaling pathway”, all three very important for both detecting pathogens/antigens and signaling for production of inflammatory cytokines and chemokines. Our results are consistent with previous studies that also observed immediate and strong proinflammatory signals and early upregulation of genes encoding acute phase proteins like chemokines, lectins and complement factors [42, 81, 82].

Evaluation of specific antibody response in the serum of vaccinated fish revealed a robust overall increase of specific antibody when comparing 62 days after vaccination against non-immunized fish, but we did not find any difference between groups, neither by strain nor diet. Dietary effects have previously been documented on antibody production or T-cell mediated responses in birds [83–85] and mammals [86–88]. The general tendency is that higher levels of n-3 PUFA improve specific immune responses in animals but the effect is species and antigen dependent. In some cases, very high dietary n-3 levels (7% of FA) may have negative effects on antibody responses [84]. There are few studies in the literature were Atlantic salmon antibody responses have been analyzed as a function of dietary FAs. Metochis et al. (2016) [89] tested total IgM in Atlantic salmon vaccinated against *A*. *salmonicida* which received different amounts of soy protein. They showed different levels in total IgM between naïve and immunized fish, but no difference among the dietary groups were observed. Atlantic salmon therefore seems to be fairly robust against detrimental effects on humoral immune responses when fed low levels of long chain n-3 PUFA. It was therefore in agreement between the early analysis of innate immune responses (24 h head kidney transcriptome) and late analysis of adaptive immune responses (62 days vaccine-specific IgM levels). If these results can be verified at the individual animal level (by analyzing blood cell transcriptome) in the same fish, as later subjected to IgM ELISA, a protocol for systems immunology analysis of salmonids can be developed. Identification of early surrogate markers of protective immune responses in aquaculture species will greatly facilitate development of new and improved vaccines [90].

The n-3 levels tested in this study were below the levels previously shown to inhibit the salmon immune system [8], but still above the daily requirement for proper immune function in controlled experimental conditions. Bou and collaborators (2017) [29] showed that the levels of EPA and DHA considered sufficient, in experimental conditions, were too low to maintain fish health or robustness when fish was kept in sea cages under commercial conditions. Another study in gilthead sea bream (*Sparus aurata L*.) found no effect of diet (up to 66% of vegetable oil) on gut transcriptome, but when the fish were challenged with *Enteromyxum leei*, significant alterations of immune related gene expression were observed [91]. Our results shows that Atlantic salmon is capable to stay healthy and to mount immune response after vaccination, in experimental conditions, even with low dietary levels of long-chain omega-3 fatty acids, but it is important to test new feed formulations in actual commercial conditions and challenging the fish with pathogens.

## Supporting information

S1 Text Supplementary information. (DOCX)

S1 Script Exploratory analysis script. (RMD)

S2 Script Transcriptome analysis script. (R)

S1 Table All expressed genes in head kidney of vaccinated fish. (XLSX)

S2 Table Differentially expressed genes in response to vaccine in head kidney. (XLSX)

S3 Table Gene ontology analysis of 442 up/downregulated differentially expressed genes induced by vaccine. (XLSX)

S4 Table KEGG analysis of the 442 up/downregulated differentially expressed genes induced by vaccine. (XLSX)

S5 Table Differentially expressed genes in head kidney of vaccinated fish using strain as covariate. (XLSX)
