# Supplementary material for: Interaction between dietary fatty acids and genotype on immune response in Atlantic salmon (Salmo salar) after vaccination: A transcriptome study
Source: PLoS One. 2019 Jul 31;14(7):e0219625. doi: 10.1371/journal.pone.0219625 (PMC6668776; doi:10.1371/journal.pone.0219625)
Supplement: S1 Text — (DOCX) [file pone.0219625.s001.docx]

Interaction between dietary fatty acids and genotype on immune response in Atlantic salmon (*Salmo salar*) after vaccination: a transcriptome study.

Adriana Magalhães Santos Andresen, Esmail Lutfi, Bente Ruyter, Gerd Berge and Tor Gjøen

24 mai, 2019

This supplementary file provides sample information and exploratory plots for the manuscript “Interaction between dietary fatty acids and genotype on immune response in Atlantic salmon (*Salmo salar*) after vaccination: a transcriptome study”. It shows the validation of ELISA for analysis of vaccine-specific IgM production in Atlantic salmon, rnaseq mapping summary, rnaseq count data distributions, replicate correlations and sample clustering. One plot also shows QPCR data plottet with the corresponding rnaseq data for a small selection of genes. Analyses have been performed using the R statistical programming language and R Bioconductor packages (a complete list is given in the bottom of the document (Session info)). This supplementary file was generated using the knitr R package under Windows 7.

## Supplementary figure A


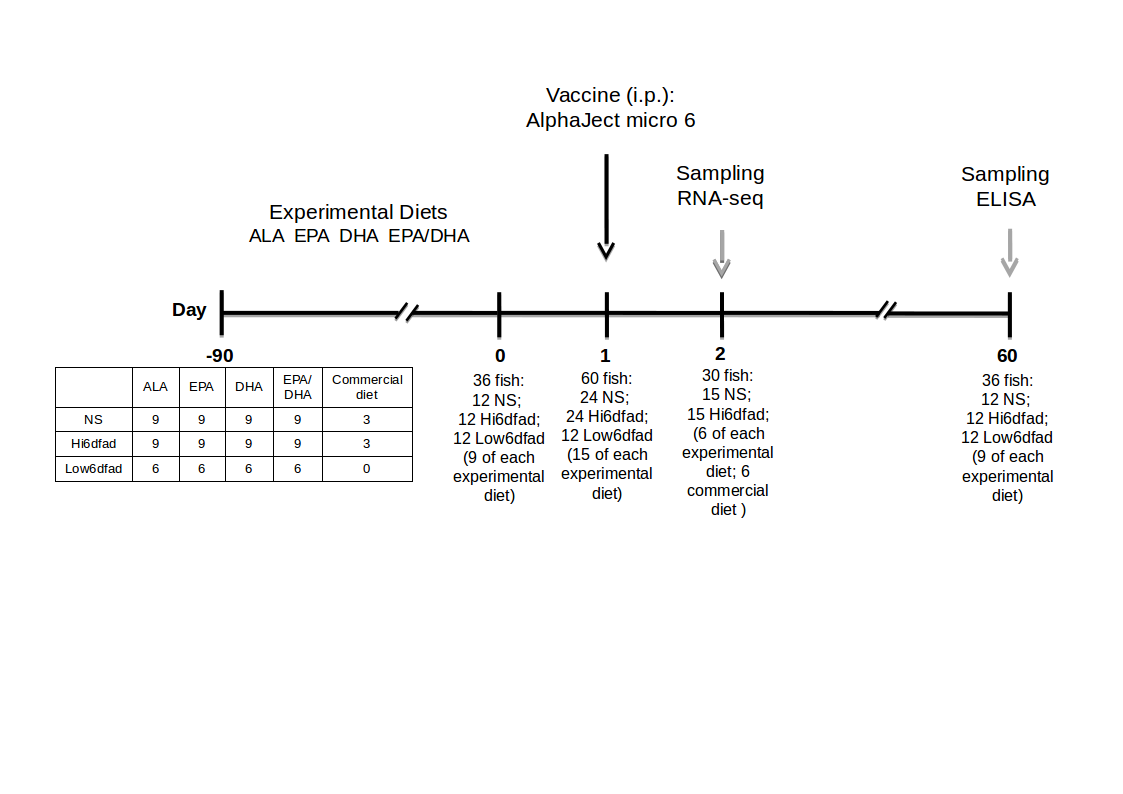


Flowchart of study design and experimental groups

## Supplementary figure B.


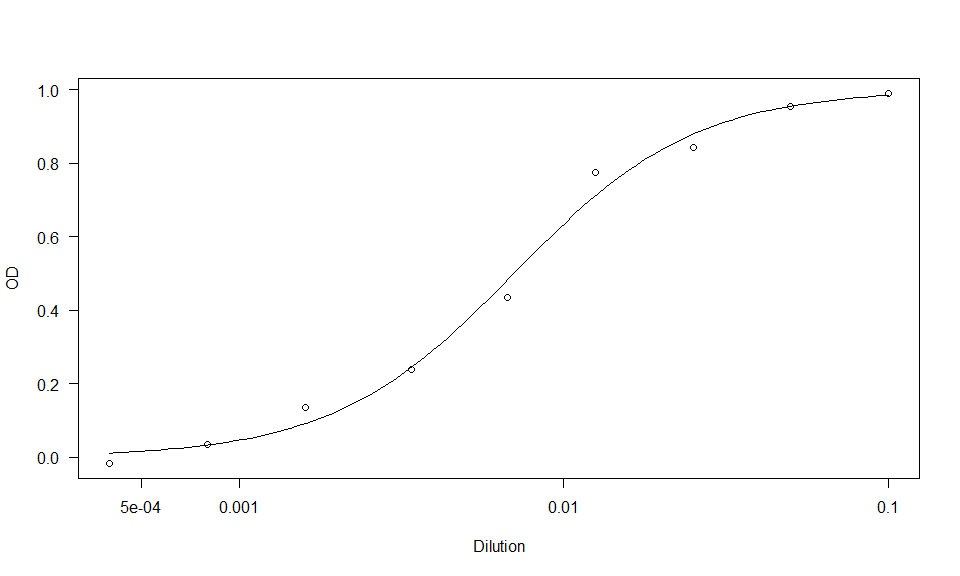


Standard curve displaying vaccine-specific ELISA OD values as a function of plasma concentration. Plasma from 10 immunized fish were mixed and serially diluted (1:2) with PBS ranging from 1:10 to 1:2560. Datapoints are mean of 8 replicates and curve show best fit using log-logistic regression (4 parameters, R-package drc).

## Supplementary table A

| id | K1CD1 | K2CD1 | K3CD1 | K1CHi1 | K2CHi1 | K3CHi1 |
| --- | --- | --- | --- | --- | --- | --- |
| MSTRG.10002 | 1.4 | 1.2 | 1.4 | 0.85 | 1.3 | 2.2 |
| MSTRG.10004 | 21 | 28 | 3.4 | 17 | 25 | 26 |
| MSTRG.10006 | 1.3 | 1.4 | 0.54 | 1.5 | 2.7 | 2.1 |
| MSTRG.10008 | 4.1 | 4 | 2.7 | 3.7 | 4.9 | 4.2 |
| MSTRG.1001 | 1.6 | 1.7 | 3.5 | 2.2 | 2.8 | 1.8 |
| MSTRG.10010 | 1.7 | 1.7 | 1.4 | 1.4 | 2.1 | 1.8 |
| MSTRG.10013 | 7.5 | 7.9 | 4.6 | 3.7 | 8.2 | 5.6 |
| MSTRG.10019 | 7.4 | 10 | 12 | 8.1 | 8.5 | 8.6 |
| MSTRG.10022 | 13 | 17 | 9.5 | 12 | 14 | 16 |
| MSTRG.10024 | 0.41 | 1.8 | 0.53 | 1.4 | 1.2 | 1.8 |

Table showing the first 10 rows / 6 samples after filtering the output from Stringtie. Genes with no annotation or no expression in head kidney were filtered, resulting in a table with a total of 3949 genes analysed for differential expression.

## Supplementary table B

| Sample | Reads | Alignment | Diet | Treatment | Strain |
| --- | --- | --- | --- | --- | --- |
| K1CD1 | 20079436 | 63.90% | Control | No_vaccine | NS |
| K1CHi1 | 24906190 | 76.51% | Control | No_vaccine | Hi6fad_b |
| K2CD1 | 21290081 | 80.97% | Control | No_vaccine | NS |
| K2CHi1 | 18610024 | 81.41% | Control | No_vaccine | Hi6fad_b |
| K3CD1 | 20858638 | 82.16% | Control | No_vaccine | NS |
| K3CHi1 | 23558734 | 81.87% | Control | No_vaccine | Hi6fad_b |
| K1DN1 | 20161562 | 82.12% | ALA | Vaccine | NS |
| K1HiN1 | 21231442 | 83.01% | ALA | Vaccine | Hi6fad_b |
| K2DN1 | 23229748 | 83.40% | ALA | Vaccine | NS |
| K2HiN1 | 23326190 | 88.50% | ALA | Vaccine | Hi6fad_b |
| K3DN1 | 27880012 | 86.44% | ALA | Vaccine | NS |
| K3HiN1 | 24481489 | 87.12% | ALA | Vaccine | Hi6fad_b |
| K1DD1 | 20623373 | 76.78% | DHA | Vaccine | NS |
| K1HiD1 | 24080928 | 85.22% | DHA | Vaccine | Hi6fad_b |
| K2DD1 | 22722861 | 86.08% | DHA | Vaccine | NS |
| K2HiD1 | 26890898 | 85.70% | DHA | Vaccine | Hi6fad_b |
| K3DD1 | 20929483 | 86.55% | DHA | Vaccine | NS |
| K3HiD1 | 26206924 | 84.98% | DHA | Vaccine | Hi6fad_b |
| K1DE1 | 23217020 | 85.92% | EPA | Vaccine | NS |
| K1HiE1 | 15952554 | 83.09% | EPA | Vaccine | Hi6fad_b |
| K2DE1 | 24553542 | 86.38% | EPA | Vaccine | NS |
| K2HiE1 | 23760713 | 84.52% | EPA | Vaccine | Hi6fad_b |
| K3DE1 | 26809649 | 79.09% | EPA | Vaccine | NS |
| K3HiE1 | 20778343 | 87.14% | EPA | Vaccine | Hi6fad_b |
| K1DED1 | 26058949 | 84.00% | EPA_DHA | Vaccine | NS |
| K1HiED1 | 24381452 | 85.42% | EPA_DHA | Vaccine | Hi6fad_b |
| K2DED1 | 24734382 | 84.77% | EPA_DHA | Vaccine | NS |
| K2HiED1 | 22415190 | 82.94% | EPA_DHA | Vaccine | Hi6fad_b |
| K3DED1 | 25211114 | 84.89% | EPA_DHA | Vaccine | NS |
| K3HiED1 | 35711928 | 81.70% | EPA_DHA | Vaccine | Hi6fad_b |

Sample names, experimental groups and alignment statistics for the groups included in the kidney transcriptome analysis. On average, 24.3 million reads were sequenced and mapped with an average frequency of 83.0 % to the Atlantic salmon genome.

## Supplementary figure C


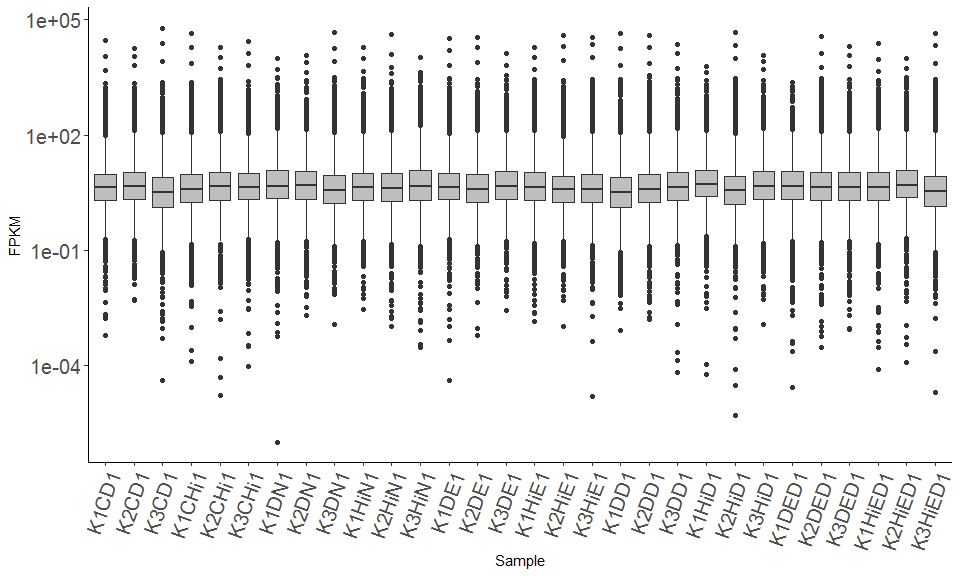


Boxplot display of the distribution of FPKM counts in each sample

# Supplementary figure D


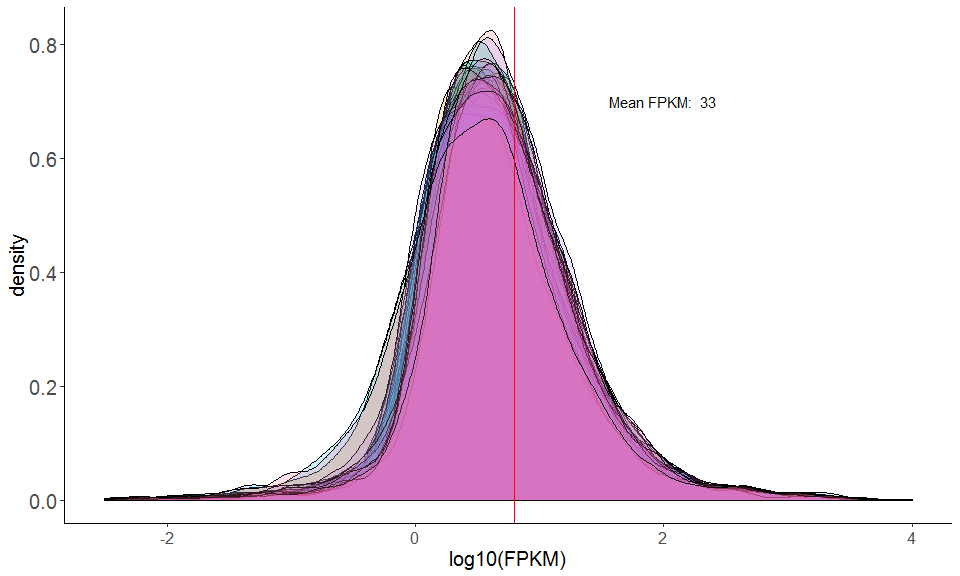


Density plot comparing the distribution of counts in each sample

## Supplementary figure E


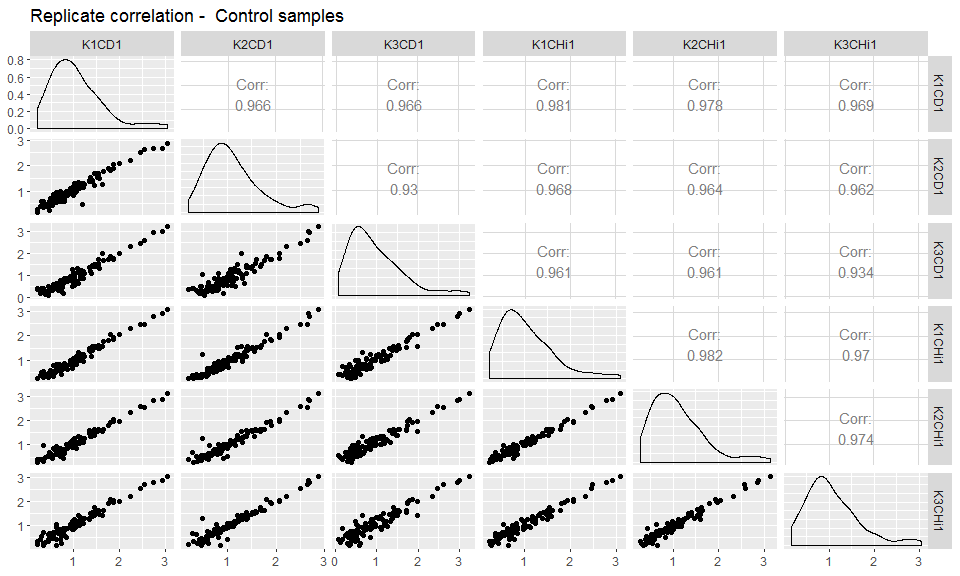

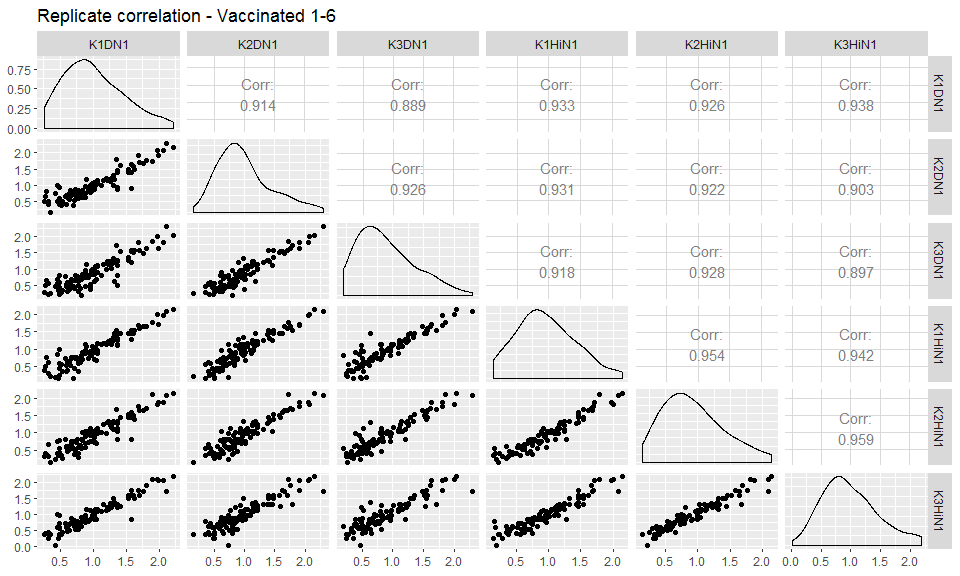


Plots showing sample data correlation between 100 randomly selected genes with at least 1 FPKM in all samples for 6 unvaccinated (upper panel) and 6 vaccinated (lower panel) fish. The panels along the diagonal display the density distribution of FPKM in the selected samples and the rigth side of the panel shows the Pearson correlation coefficient for each sample pair.

## Supplementary figure F


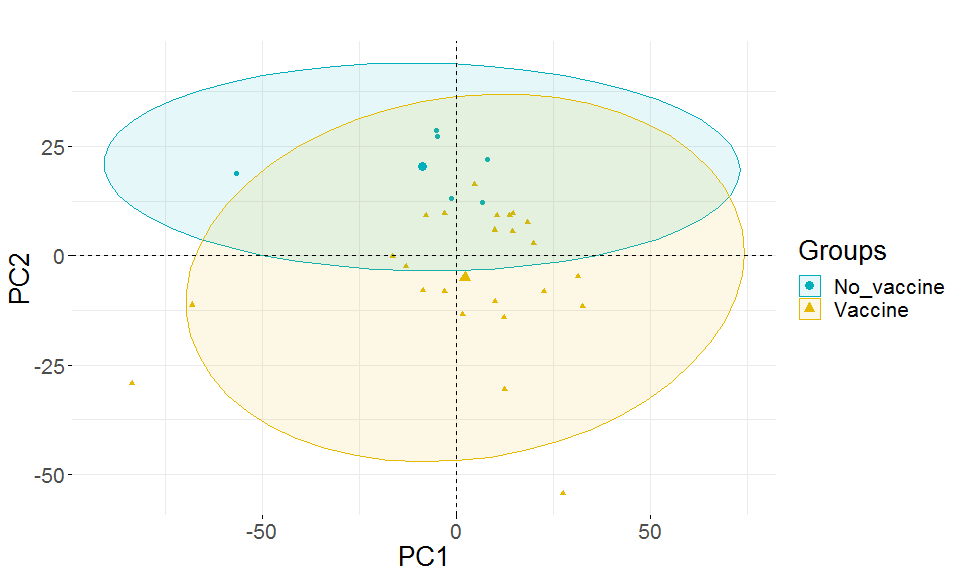


Principal component analysis of log2 transformed FPKM gene expression values (n=3949 genes) shows that most of the variation in gene expression could be assigned to vaccination with two identifiable clusters. Diet or strain did not contribute significantly to the total variation in gene expression.

## Supplementary figure G


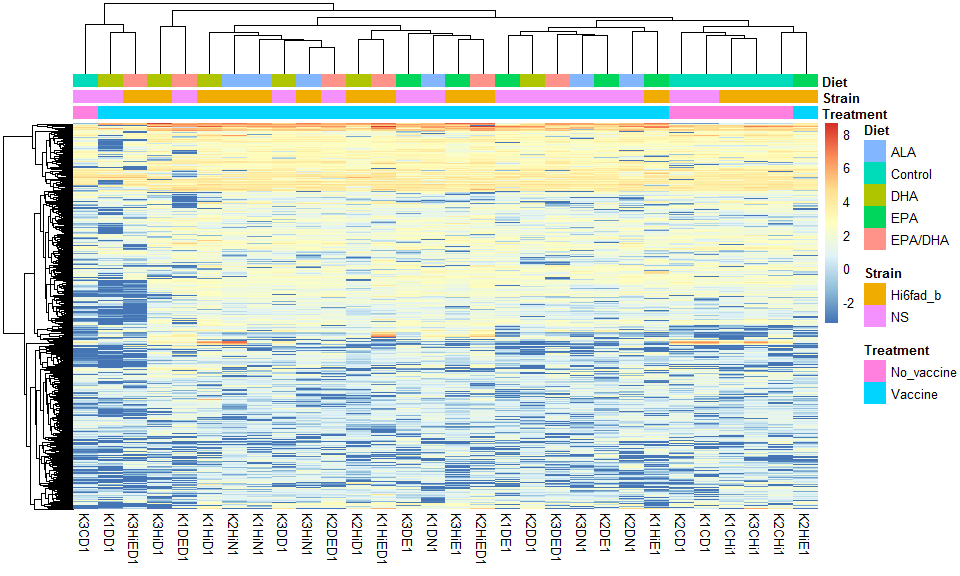


Hierarchical clustering heatmap (euclidian distance) of log2 FPKM from the 5 % most variable genes (subset containing 936 most variable genes selected by descending rowVar across 30 samples, n= 3949).

## Supplementary figure H


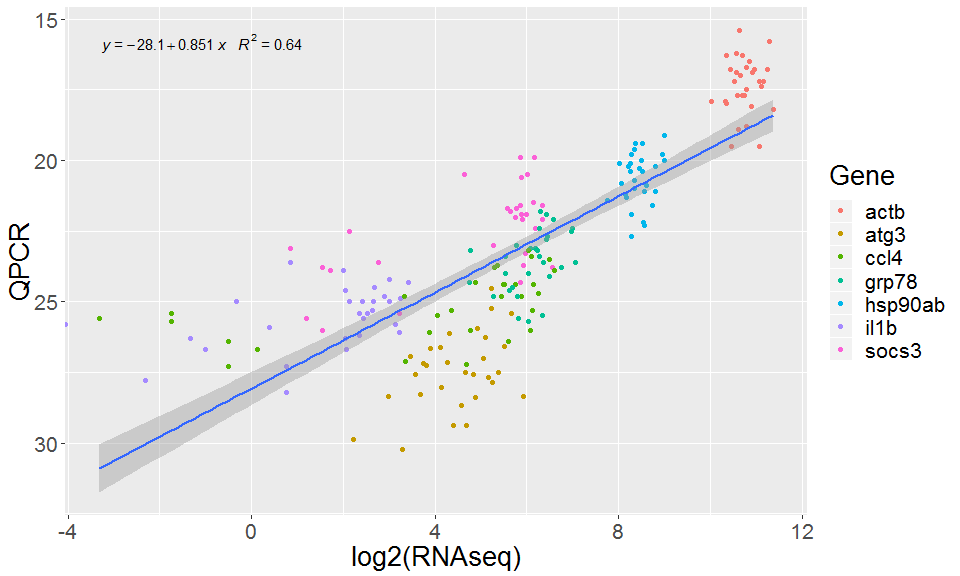


Correlation between rnaseq and QPCR analysis of selected genes from head kidney samples of vaccinated Atlantic salmon.

##Session information with list of R packages

## R version 3.5.3 (2019-03-11)
## Platform: x86_64-w64-mingw32/x64 (64-bit)
## Running under: Windows 7 x64 (build 7601) Service Pack 1
##
## Matrix products: default
##
## locale:
## [1] LC_COLLATE=Norwegian (Bokmål)_Norway.1252
## [2] LC_CTYPE=Norwegian (Bokmål)_Norway.1252
## [3] LC_MONETARY=Norwegian (Bokmål)_Norway.1252
## [4] LC_NUMERIC=C
## [5] LC_TIME=Norwegian (Bokmål)_Norway.1252
##
## attached base packages:
## [1] parallel stats4 stats graphics grDevices utils datasets
## [8] methods base
##
## other attached packages:
## [1] ggpmisc_0.3.1 pheatmap_1.0.12
## [3] readr_1.3.1 pander_0.6.3
## [5] drc_3.0-1 MASS_7.3-51.4
## [7] DESeq2_1.22.2 SummarizedExperiment_1.12.0
## [9] DelayedArray_0.8.0 BiocParallel_1.16.6
## [11] matrixStats_0.54.0 Biobase_2.42.0
## [13] GenomicRanges_1.34.0 GenomeInfoDb_1.18.2
## [15] IRanges_2.16.0 S4Vectors_0.20.1
## [17] BiocGenerics_0.28.0 genefilter_1.64.0
## [19] factoextra_1.0.5 ggfortify_0.4.6
## [21] GGally_1.4.0 dplyr_0.8.0.1
## [23] kableExtra_1.1.0 tidyr_0.8.3
## [25] reshape2_1.4.3 ggplot2_3.1.0
##
## loaded via a namespace (and not attached):
## [1] TH.data_1.0-10 colorspace_1.4-1 rio_0.5.16
## [4] htmlTable_1.13.1 XVector_0.22.0 base64enc_0.1-3
## [7] rstudioapi_0.10 ggpubr_0.2 ggrepel_0.8.0
## [10] bit64_0.9-7 AnnotationDbi_1.44.0 mvtnorm_1.0-10
## [13] xml2_1.2.0 codetools_0.2-16 splines_3.5.3
## [16] geneplotter_1.60.0 knitr_1.22 polynom_1.4-0
## [19] Formula_1.2-3 annotate_1.60.1 cluster_2.0.9
## [22] compiler_3.5.3 httr_1.4.0 backports_1.1.4
## [25] assertthat_0.2.1 Matrix_1.2-17 lazyeval_0.2.2
## [28] acepack_1.4.1 htmltools_0.3.6 tools_3.5.3
## [31] gtable_0.3.0 glue_1.3.1 GenomeInfoDbData_1.2.0
## [34] Rcpp_1.0.1 carData_3.0-2 cellranger_1.1.0
## [37] xfun_0.6 stringr_1.4.0 openxlsx_4.1.0
## [40] rvest_0.3.3 gtools_3.8.1 XML_3.98-1.19
## [43] zlibbioc_1.28.0 zoo_1.8-5 scales_1.0.0
## [46] hms_0.4.2 sandwich_2.5-1 RColorBrewer_1.1-2
## [49] yaml_2.2.0 curl_3.3 memoise_1.1.0
## [52] gridExtra_2.3 rpart_4.1-15 reshape_0.8.8
## [55] latticeExtra_0.6-28 stringi_1.4.3 RSQLite_2.1.1
## [58] plotrix_3.7-5 checkmate_1.9.1 zip_2.0.1
## [61] rlang_0.3.4 pkgconfig_2.0.2 bitops_1.0-6
## [64] evaluate_0.13 lattice_0.20-38 purrr_0.3.2
## [67] labeling_0.3 htmlwidgets_1.3 bit_1.1-14
## [70] tidyselect_0.2.5 plyr_1.8.4 magrittr_1.5
## [73] R6_2.4.0 Hmisc_4.2-0 multcomp_1.4-10
## [76] DBI_1.0.0 pillar_1.3.1 haven_2.1.0
## [79] foreign_0.8-71 withr_2.1.2 survival_2.44-1.1
## [82] abind_1.4-5 RCurl_1.95-4.12 nnet_7.3-12
## [85] tibble_2.1.1 crayon_1.3.4 car_3.0-2
## [88] rmarkdown_1.12 locfit_1.5-9.1 grid_3.5.3
## [91] readxl_1.3.1 data.table_1.12.2 blob_1.1.1
## [94] forcats_0.4.0 digest_0.6.18 webshot_0.5.1
## [97] xtable_1.8-4 munsell_0.5.0 viridisLite_0.3.0
